# The WHO FCTC and global governance: effects and implications for future global public health instruments

**DOI:** 10.1136/tobaccocontrol-2018-054358

**Published:** 2018-09-14

**Authors:** Thomas F McInerney

**Keywords:** global health, globalisation, public policy

## Abstract

**Introduction:**

This article analyses experience with the WHO Framework Convention on Tobacco Control (WHO FCTC) in the context of global governance. It examines ways in which the WHO FCTC has been addressed by international institutions, particularly within the UN system at the international and national levels. It seeks to understand the extent to which the WHO FCTC as an international legal obligation has contributed to its integration in the strategies, policies and programmes of UN system organisations.

**Methods:**

The article examines documentation reflecting global governance responses to tobacco control since the Convention’s entry into force in 2005. It also considers discussions with officials involved in the design and management of various UN initiatives. Finally, it draws on the findings of the Expert Group on the Impact Assessment of the WHO FCTC.

**Conclusions:**

The influence of the WHO FCTC in global governance can be at least partially attributed to its status as an international legal obligation. While tobacco control would have likely been a priority in international public health even in the absence of the WHO FCTC, the importance of tobacco control has been relatively greater as a result of the treaty. In assessing the potential utility of any future global public health instrument, it is important to consider the need to mobilise action by other global governance institutions and ensure that the chosen instrument will be capable of stimulating such action.

## Introduction

Experience with the WHO Framework Convention on Tobacco Control (WHO FCTC) illustrates the dynamics in global governance processes whereby efforts of treaty secretariats and international organizations combine with political decisions of member states, advocacy by civil society, and philanthropic support to advance the agenda of the treaty.

To understand these questions, the article will examine documentation reflecting global governance responses to tobacco control since the Convention’s entry into force in 2005 including the Strategic Plan for the WHO, the development of the Inter-agency Task Force on Noncommunicable Disease, the UN Sustainable Development Goals (SDGs) and United Nations Development Group guidelines for United Nations Development Assistance Frameworks. It also benefited from discussions with officials involved in the design and management of these initiatives. Finally, it draws on the findings of the report of the Expert Group on the Impact Assessment of the WHO FCTC. 

Previous authors have identified a series of additions to the global health architecture following the enactment of the WHO FCTC.^1^ These include the creation of dedicated functions, notably secretariats, to perform executive functions for the WHO FCTC and the Protocol to Eliminate Illicit Trade in Tobacco Products (Protocol). Under the Convention, further actions have been taken to support its implementation and adherence by parties. These measures include the various guidelines relating to substantive articles of the Convention, the reporting framework and the Protocol itself. Furthermore, the WHO FCTC has enabled the development of national legislation that was of a more rigorous character than traditional measures on the same topics.

These developments are important manifestations of the WHO FCTC’s role in global governance; however, this article focuses not on developments within the WHO FCTC itself but instead coordination within the UN system and among other international actors.

## Background

To understand the challenges involved and approaches taken to integrating the WHO FCTC in global governance, it is necessary to first consider its legal status. The WHO FCTC is an international legal agreement or treaty, which is subject to international law. Under international law, treaties are only legally binding on the states that sign and ratify them. International organisations, such as WHO, are created through treaties but do not exercise any legal authority over other treaties. Each treaty is thus legally autonomous. The Protocol is a treaty in its own right but may only be signed by parties to the WHO FCTC.^2^

Taking into account the separate juridical status of international agreements and international organisations is important to understand the relationship between the WHO FCTC and the WHO. Even though the Convention was developed under the auspices of the WHO and was approved by the World Health Assembly (WHA) on 21 May 2003, the WHO has no direct legal authority over the WHO FCTC or its operations. The WHO FCTC is hosted by WHO in its headquarters but is not a function, department or unit of WHO under its control. Moreover, the governing body of the FCTC, the Conference of the Parties (COP), is independent of the WHA. Despite this separateness, WHO has taken significant steps to advance the WHO FCTC.

Beyond the WHO, gaining support for the FCTC among other international actors, particularly within the UN system, is not a straightforward matter. The UN system is akin to a federation rather than a hierarchy. While the Secretariat of the UN falls under the authority of the Secretary General and General Assembly, the specialised agencies (eg, WHO or Food and Agriculture Organization (FAO)) and programmes (eg, the UN Development Programme (UNDP)) have their own separate governing bodies. Outside actors have limited ability to affect decisions of those bodies and practices of the organisations. Institutional fragmentation and policy incoherence resulting from this structure have bedevilled the UN system for decades. In this regard, support for the WHO FCTC by other UN agencies, programmes, and departments depends on voluntary action of those entities.

## Global governance efforts to support WHO FCTC implementation

Since it entered into force in 2005, the WHO and WHA have taken a series of actions to support the ratification and implementation of the WHO FCTC directly and indirectly as part of broader non-communicable disease (NCD) response. These include the prioritisation of the WHO FCTC by the WHO, giving prominence to the WHO FCTC in the tobacco control-related elements of the UN’s broad NCD agenda, and the inclusion of the WHO FCTC in the 2030 Sustainable Development Agenda and SDGs. Within the WHO, efforts have been made to support the WHO FCTC, among which were the inclusion of the WHO FCTC in the WHO Strategic Plan, prioritising the Convention in its response to NCDs, carrying out programmes through the WHO’s Tobacco Free Initiative (TFI), particularly the launch of the MPOWER initiative to promote implementation of specific elements of the WHO FCTC.

In reviewing these developments, it is important to note that the efforts of the WHO and civil society to advance the FCTC have been significantly enhanced through approximately US$1 billion in funding from Bloomberg Philanthropies and the Bill and Melinda Gates Foundation. These private philanthropic actors have supported a range of initiatives, including direct support for WHO and what they refer to as ‘best practice’^3^ or ‘proven tobacco control policies’^4^ from the WHO FCTC, notably MPOWER. In the press release announcing Bloomberg’s additional pledge of US$360 million, Uruguayan President Dr Tabaré Vásquez said the funding came ‘at a critical time in order to help countries implement’ the WHO FCTC.^5^ The fact that so much of the funding by these philanthropies has gone to support measures in the WHO FCTC makes it an important factor to consider in measuring the treaty’s effectiveness.

An important step towards supporting WHO FCTC implementation was the WHO’s Medium Term Strategic Plan (MTSP) for 2008–2013, which prioritised WHO FCTC implementation. Strategic Objective 6.3 provided technical support to address the needs of Member States with high burdens of disease and death from tobacco use and respond to relevant public health institutional needs while supporting the COP to the WHO FCTC to implement the Convention’s provisions and develop and implement guidance materials and protocols.

In addition to the MTSP of WHO, the WHO Global Action Plan for the Prevention and Control of Noncommunicable Diseases (NCD Action Plan) has prioritised tobacco control. Under objective 3, the NCD Action Plan seeks to ‘Reduce modifiable risk factors for Noncommunicable Diseases and underlying social determinants through creation of health-promoting environments’.^6^ Among the policy options for achieving this objective in respect of tobacco control, the document calls for accelerating implementation of the WHO FCTC among WHO member states, which are parties to the Convention and having non-parties consider joining.

As a programme of WHO, TFI is independent of the WHO FCTC. The TFI undertakes capacity building, technical assistance and surveillance that support states’ adherence to the WHO FCTC generally, as well as promoting specific articles. In terms of specific articles, the WHO has developed the Bloomberg-funded MPOWER initiative with support from Bloomberg Philanthropies, which advances measures that ‘support scale up of specific provisions of the WHO FCTC on the ground’.^7^ While MPOWER has prioritised six of the WHO FCTC articles—a move that has elicited some concern among parties, the WHO FCTC Secretariat and other stakeholders—from the standpoint of global governance, it has had the effect of aligning WHO tobacco control efforts with the WHO FCTC.

Outside of the WHO, efforts have been made to prioritise the WHO FCTC among other global governance institutions and activities. Key among these is the treaty’s inclusion as a target in the SDGs. This action will have significant consequences. Initial experience suggests that the 2030 Agenda and SDGs will shape the response of the entire UN system, bilateral development agencies, multilateral development agencies, philanthropy, NGOs and private sector for 15 years. SDG Target 3a calls to ‘strengthen the implementation of the World Health Organization Framework Convention on Tobacco Control in all countries, as appropriate’.^8^

To put this approach in context, it is important to recognise that the SDGs implicitly give effect to many multilateral treaties covering diverse fields but explicitly mention only a few. As an example, the three chemical conventions (Basel Convention, Stockholm Convention and Rotterdam Convention) are not referenced by name but instead implicitly recognised in relation to SDG targets 3.9, 6.3, and 12.4 relating to chemical use and waste.^8^

The decision to make the WHO FCTC a target of the SDGs stands in contrast to the situation with the Millennium Development Goals (MDGs), which were in place from 2000 to 2015. Tobacco control was not referenced in the MDGs, while three other health objectives (MDG 4 aims to reduce child mortality, MDG 5 aims to improve maternal health and MDG 6 aims combat HIV/AIDS, malaria and other diseases) were identified.^9^ A result of this situation was that countries prioritised those MDGs in their national development strategies, which required efforts to raise the profile of tobacco control vis-a-vis these other concerns.

It is likely that the UN might have made tobacco control a target for the SDGs had the WHO FCTC not been in force; however, the target that was created quite clearly speaks to the treaty itself. The efforts of the treaty secretariat, parties and other stakeholders to give prominence to the WHO FCTC in the SDGs were obviously successful. Given the number of actors and interests involved in the process of developing the SDGs, the inclusion of WHO FCTC implementation as a target is some indication of its status as an international obligation. This step suggests that UN members recognised the importance of all of the obligations to undertake tobacco control entailed by the WHO FCTC.

In addition to global governance measures directly supporting the WHO FCTC, the broader UN response to NCDs has created additional support for the WHO FCTC. The UN has made NCDs a major priority with the UN General Assembly, holding two high-level meetings on NCDs in 2011 and 2014 with a third planned for 2018. The WHO FCTC has been central to the development of the UN response to NCDs and is a major element of it.

Dissociating the tobacco-related elements of the broader NCD agenda from its other priorities is difficult. It is, however, clear that the NCD agenda might never have begun had there not been the normative commitment of the international community in the form of the WHO FCTC. Without it, precedent for normative global health instruments would have been relatively weaker. Moreover, given that tobacco use was recognised in the 2011 UN Political Declaration on NCDs as a leading cause of preventable deaths per year, it is understandable that a significant amount of the emphasis for the NCD agenda relates to the WHO FCTC.

Overall, the relationship between the WHO FCTC and the broader NCD agenda is mutually supportive. The Political Declaration on NCDs adopted in 2011 called for accelerating implementation of the WHO FCTC, while recognising ‘that substantially reducing tobacco consumption is an important contribution to reducing non-communicable diseases’.^10^

Synergies between the WHO FCTC and the NCD agenda are also evident in the reference in the Addis Ababa Action Agenda of the Third International Financing for Development Conference in 2015, which specifies means of financing achievement of the 2030 Agenda and SDGs. In the context of addressing the costs of NCDs for national budgets due to the burden of NCDs on healthcare systems, the UN member states agreed on the following statement:

We note the enormous burden that non-communicable diseases place on developed and developing countries. These costs are particularly challenging for small island developing States. We recognize, in particular, that, as part of a comprehensive strategy of prevention and control, price and tax measures on tobacco can be an effective and important means to reduce tobacco consumption and health care costs, and represent a revenue stream for financing for development in many countries.^11^

Accordingly, UN member states recognised that taxes on tobacco can be useful to address the healthcare costs incurred because of tobacco use and those attributable to non-tobacco related NCDs and broader development efforts. The effort to include this language in the financing for development agenda was led by the WHO FCTC Secretariat, member states and civil society. Here, too, the status of the WHO FCTC and the numerous actors dedicated to supporting it played an important role in the outcome.

The WHO FCTC has also been a prominent element of the work of the NCD Task Force. The Task Force states in its brochure that ‘a key priority for the Task Force is to ensure that Member States can harness the leadership and support of the whole UN system for implementing the WHO Framework Convention on Tobacco Control’. Furthermore, it states that ‘tobacco control is a major component of joint programming missions’.^12^

The Task Force includes more than 40 UN agencies and departments. Initially, the WHO FCTC Secretariat was represented by WHO; however, in recognition of their respective roles in tobacco control, the WHO FCTC Secretariat became a member in its own right. The fact that both the WHO and the Secretariat of the WHO FCTC are members has helped ensure that the WHO FCTC receives attention by the group.

The Task Force draws support from the UN agencies and from UN member states themselves. The Friends of the Task Force is an informal group of UN member states, which support the NCD Task Force both financially and programmatically. It is important to recognise that not all interagency processes in the UN have such systems in place. Hence, this situation reflects a degree of support by UN members for its mission.

Among the elements of the 2016–2017 Work Program of the Task Force is an effort to create a consistent UN system-wide approach to addressing tobacco industry interference. Grounds for this approach were set in the 2011 NCD Political Declaration, which recognised the ‘fundamental conflict of interest between the industry and public health’.^10^ In line with this understanding, the Work Program calls for reviewing ‘policies and practices among UN agencies on interaction with and potential interference from tobacco industry and its front group’.^9^ Following this statement, the Task Force and WHO FCTC Secretariat put forward a draft policy on tobacco industry interference for use by all UN agencies, departments and programmes.^13^

UN member states have since expressed their support for the draft policy. In 2017, United Nations Economic and Social Council  (ECOSOC) approved a resolution to address NCDs, which referenced the member states’ consensus in support for efforts to prevent tobacco industry interference. The resolution ‘encourages members of the Task Force, as appropriate and in line with their respective mandates, to develop and implement their own policies on preventing tobacco industry interference, bearing in mind the model policy for agencies of the United Nations system on preventing tobacco industry interference, in order to ensure a consistent and effective separation between the activities of the United Nations system and those of the tobacco industry’.^14^

The Task Force has also made tobacco control one of its strategic objectives. Priorities include the development of tools to cost WHO FCTC implementation (WHO, World Bank, FCTC Secretariat and UNDP), engaging with finance ministers of five countries to improve and increase taxes on tobacco products (WHO, World Bank, FCTC Secretariat and International Monetary Fund), training midwives in three countries on implementing WHO recommendations for prevention and management of tobacco use and secondhand smoke exposure during pregnancy (Unicef and United Nations Population Fund (UNFPA)), promote implementation of plain packaging among WHO FCTC parties (FCTC Secretariat WHO, United Nations Conference on Trade and Development (UNCTAD) and UNDP), promote ratification and entry into force of the Protocol to Eliminate Illicit Trade in Tobacco Products (FCTC Secretariat, World Customs Organization (WCO), World Bank and United Nations Office on Drugs and Crime (UNODC)), promoting south–south cooperation on alternative livelihoods for tobacco farmers (FCTC Secretariat, WHO, FAO, UNDP, WB, Unicef and United Nations Environmental Programme (UNEP)), establishing national multisectoral coordination mechanisms for WHO FCTC implementation in three countries (FCTC Secretariat, UNDP and WHO). To further strengthen collective efforts to implement the WHO FCTC, the Task Force has prepared a matrix of work among its members.

Aside from these efforts to coordinate support for the FCTC, there are other examples of how its status as an international legal instrument has affected decisions of various international committees and tribunals. The Committee on the Rights of the Child has referred to states’ obligations to ‘introduce into domestic law, implement and enforce internationally agreed standards concerning children’s right to health, including’ among other things the WHO FCTC.^15^ Likewise, in recommendations to Argentina, the Committee on the Elimination of Discrimination Against Women took issue with tobacco marketing directed to women and urged Argentina to ratify and implement the WHO FCTC.^16^ In addition to these human rights decisions, the FCTC was recognised as justifying action by Uruguay to adopt plain packaging. Similarly, in a dispute between the USA and Indonesia over a US effort to curtail clove cigarette sales as a way of reducing youth smoking, the World Trade Organization (WTO) Panel found that, even though neither the USA nor Indonesia had ratified the WHO FCTC, the Partial Guidelines for Implementation of Articles 9 and 10 of the Convention (Partial Guidelines) supported the US action. The tribunal recognised that from a public health perspective, there could be no justification for adding flavouring agents to cigarettes that could have the effect of making them more attractive. Citing that the Partial Guidelines drew on ‘best available scientific evidence and experience of the parties’, the US efforts could be justified because they could make a material contribution to reducing youth smoking.^17^

## Implications of the WHO FCTC experience for global public health instruments

Experience with the WHO FCTC has spawned interest in the potential for the development of other binding global public health legal instruments.^1^ Demand for further international instruments can be expected as an outgrowth of ‘globalization and the growing impact of transnational factors on health’.^1^

Treaties can facilitate and catalyse international efforts to address specific problems. They provide visibility, enable clear and consolidated statements of relevant norms and enjoy a measure of legitimacy in relation to other priorities that may tilt the scale in favour of their purposes. Despite these evident advantages, not all topics relevant to global public health are amenable to being addressed by treaties.

A key requirement for developing a treaty is political support. States must decide that an issue can be addressed more effectively through a treaty instrument than other modalities. Assuming the political support emerges, there must also be some commitment of the parties to the treaty to provide some predictable financial support for the efforts.

Experience with the FCTC shows the critical role of funding to support implementation of treaty obligations. There are many examples of treaties lacking resources that generate minimal results. The philanthropic support generated by Bloomberg and Gates has been an essential element of WHO FCTC successes, a result that might not be replicated for other instruments.

A further consideration to be anticipated in developing future instruments on global public health is whether synergies can be established between agreements. Hypothetically, if additional treaties to address NCDs were developed, a question that would arise is how those agreements might interact with the WHO FCTC. Experience in this regard in other contexts is mixed. Most often states have simply created additional instruments and subsequently have taken measures to achieve coherence.

A significant question that the parties to any future public health treaties should consider would be whether, rather than just creating a new instrument alongside the WHO FCTC, efforts to achieve coherence could be undertaken at the outset. What those might be would depend on a variety of factors.

One challenge that any future public health treaty may need to address is the question of how to address industry involvement. The WHO FCTC’s approach to preventing tobacco industry interference with tobacco control measures as provided in Article 5.3, and the associated guidelines can be contrasted with the approach taken with industry in relation to other NCDs notably food and alcohol.^18^ The WHO FCTC ‘embodies a commitment to rejecting the possibility of collaboration with the tobacco industry and an active policy of minimising interactions and policy engagement with it’.^18^ In contrast, efforts to address the harmful effects of food and alcohol have involved forms of collaboration between WHO and other UN agencies and the industry. This type of ‘tobacco exceptionalism’ (as Collin terms it) in global public health regulation, complicates—if not impedes—synergies with other global public health instruments.

One can view these different approaches as the product of overzealousness of tobacco control measures or, alternatively, insufficient caution in addressing the food and alcohol industries. As Collin has observed, the approach of the WHO FCTC to the industry contrasts sharply with the predominant practice in many other areas of regulation today. At the same time, the numerous examples of industry attempting to avoid regulation or opposing regulation for the public health including in relation to food and alcohol make it difficult to view the activities of the tobacco industry as *sui generis*. Whether it merely differs in degree or in kind is a matter to be considered in developing any future international public health instruments.

## Conclusion

This article finds persuasive evidence that the influence of the WHO FCTC in global governance can be at least partially attributed to its status as an international legal obligation. First, it finds substantial support for the proposition that the WHO FCTC has influenced the manner in which global governance institutions have incorporated tobacco control in their work. Second, it finds significant evidence for the view that the WHO FCTC may have influenced the decisions of UN system organisations to address tobacco control as a priority and to undertake measures to advance specific elements of the WHO FCTC such as Articles 5.3 and 6. Moreover, the WHO FCTC has spurred the development of the NCD agenda and constituted a major element of the UN response to NCDs. These findings suggest that, while tobacco control would have likely been a priority in international public health even in the absence of the WHO FCTC, the importance of tobacco control has been relatively greater as a result of the treaty.

Based on these findings, the article concludes that in assessing the potential utility of any future legally binding global public health instrument, it is important to consider the need to mobilise action by other global governance institutions and take steps to ensure that the chosen instrument will be strong enough to stimulate such action.

**What this paper adds**

- Previous authors have identified a series of additions to the global health architecture following the enactment of the WHO Framework Convention on Tobacco Control (WHO FCTC), including the creation of dedicated functions, notably secretariats, to perform executive functions for the WHO FCTC and Protocol on Illicit Trade in Tobacco Products, as well further actions to support implementation of the treaty, such as the development of implementation guidelines and the reporting framework.
- This paper adds to the literature on the WHO FCTC’s role in global governance by focusing not on developments within the WHO FCTC itself but instead coordination within the UN system and among other international actors.
- The paper finds persuasive evidence that the influence of the WHO FCTC in global governance can be at least partially attributed to its status as an international legal obligation. The importance of tobacco control has been relatively greater as a result of the treaty.
- It demonstrates that the WHO FCTC has influenced the manner in which global governance institutions have incorporated tobacco control in their work, may have influenced the decisions of UN system organisations to address tobacco control, has spurred the development of the non-communicable disease (NCD) agenda and has constituted a major element of the UN response to NCDs.
